# Complete chloroplast genomes of *Rubus* species (Rosaceae) and comparative analysis within the genus

**DOI:** 10.1186/s12864-021-08225-6

**Published:** 2022-01-06

**Authors:** Jiaojun Yu, Jun Fu, Yuanping Fang, Jun Xiang, Hongjin Dong

**Affiliations:** 1 Hubei Key Laboratory of Economic Forest Germplasm Improvement and Resources Comprehensive Utilization, Huanggang Normal University, Huanggang, Hubei China; 2 Hubei Collaborative Innovation Center for the Characteristic Resources Exploitation of Dabie Mountains, Huanggang Normal University, Xingang Road No. 146, Huanggang, 438000 Hubei China

**Keywords:** *Rubus*, Chloroplast genome, Compare analysis, Phylogeny

## Abstract

**Background:**

*Rubus* is the largest genus of the family Rosaceae and is valued as medicinal, edible, and ornamental plants. Here, we sequenced and assembled eight chloroplast (cp) genomes of *Rubus* from the Dabie Mountains in Central China. Fifty-one *Rubus* species were comparatively analyzed for the cp genomes including the eight newly discovered genomes and forty-three previously reported in GenBank database (NCBI).

**Results:**

The eight newly obtained cp genomes had the same quadripartite structure as the other cp genomes in *Rubus*. The length of the eight plastomes ranged from 155,546 bp to 156,321 bp with similar GC content (37.0 to 37.3%). The results indicated 133–134 genes were annotated for the *Rubus* plastomes, which contained 88 or 89 protein coding genes (PCGs), 37 transfer RNA genes (tRNAs), and eight ribosomal RNA genes (rRNAs). Among them, 16 (or 18) of the genes were duplicated in the IR region. Structural comparative analysis results showed that the gene content and order were relatively preserved. Nucleotide variability analysis identified nine hotspot regions for genomic divergence and multiple simple sequences repeats (SSRs), which may be used as markers for genetic diversity and phylogenetic analysis. Phylogenetic relationships were highly supported within the family Rosaceae, as evidenced by sub-clade taxa cp genome sequences.

**Conclusion:**

Thus, the whole plastome may be used as a super-marker in phylogenetic studies of this genus.

**Supplementary Information:**

The online version contains supplementary material available at 10.1186/s12864-021-08225-6.

## Background

*Rubus* is the largest genus in the family Rosaceae, with approximately 700 species. Plants are widely distributed in the temperate and sub-tropical regions of the Northern Hemisphere, with only a few species found in the Southern Hemisphere. There are more than 208 species found in China, 139 of which are endemic [1]. There is a growing interest in the abundance of regional taxonomic treatments, new species, and new accounts of the genus *Rubus* in China [2–12].

*Rubus* has been used in traditional Chinese medicine since ancient times. The fruits are well-known in Asia, Europe, and North America and have a long history of usefulness in these regions, especially in some European countries. Some natural species, such as *R. corchorifolius*, are widely cultivated for their medicinal, edible, and ornamental value.

*Rubus* is well-known for its diversity, leaf shape, inflorescence architecture, reproductive pattern, and other features. The plant is typically armed with bristles, prickles, or glandular hairs; the leaf exhibits simple, palmate, or pinnate shapes; flowers are pentamerous and mostly bisexual; achenes are drupelets or drupaceous and are aggregated on a penduncle as semispherical, conical, or cylindrical fruits [1]. *Rubus* is one of the most difficult taxa of flowering plants to classify due to its highly variable morphologies, and its complicated apomixis, polyploidy, and hybridization [13, 14]. Therefore, it is essential to construct a phylogeny tree using molecular evidence to better understand the relationships between species and improve the development and utilization of wild germplasm resources.

The most widely-accepted taxonomic system of the genus *Rubus* was built by Focke [15–17]. According to Focke, *Rubus* was separated into 12 subgenera, the largest being *Rubus* (132 species), *Idaeobatus* (117 species), and *Malachobatus* (115 species). *Rubus* is found mainly in Europe and North America, according to Flora of China (FOC), while *Malachobatus* and *Idaeobatus* are typically found throughout Asia, especially China [1].

Several molecular phylogenetic studies have tried to resolve the genetic relationships of *Rubus* [13, 18–22]. Wang et al. (2016) selected three chloroplasts (*rbcL*, *rpl20*-*rps12*, and *trnG*-*trnS*) and three nuclear genetic markers (nrITS, *GBSSI*-2, and *PEPC*) to resolve the phylogenetic relationships of 142 Chinese *Rubus* species, in which the phylogeny showed a certain degree of inadequacy between the chloroplast and nuclear markers.

There are few reports on the chloroplast genomes of *Rubus* to date [23–30]. Recently, a comparative analysis of the characteristics of the *Rubus* cp genome was reported, and together with about other ten species was used to construct a phylogenetic tree, but the mainly species was distributed in Taiwan [31]. Here, we sequenced and assembled eight cp genomes of *Rubus* and comparatively analyzed. And together with previously reported 38 cp genomes downloaded from the organelle genome database at National Center for Biotechnology Information (https://www.ncbi.nlm.nih.gov) [32], phylogenetic analysis was also performed. Our results, including gene content, size, nucleotide variable sites, identified SSRs, and phylogeny analysis, may improve our understanding of the cp genomes structure of genus *Rubus* and provide resources for genetic diversity and phylogenetic analyses in future studies.

## Results and discussion

### General features of Rubus chloroplast genomes

**Fig. 1:**
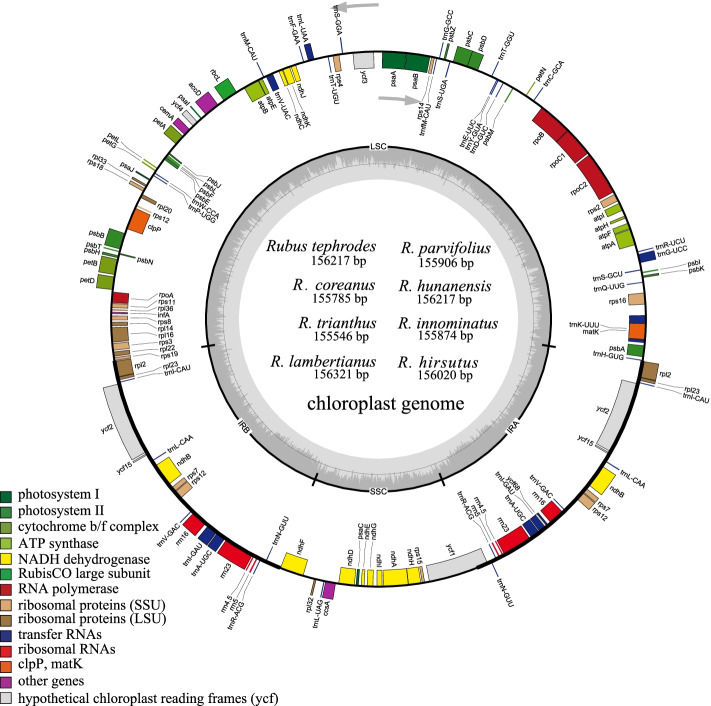
Gene maps of the complete cp genome of eight species of *Rubus*. Genes on the inside of the circle are transcribed clockwise, while that outside are transcribed counter clockwise. Genes were colored according to their functional groups. The darker gray in the inner circle corresponds to GC content, whereas the lighter gray corresponds to AT content

For the eight newly sequenced species, Illumina PE sequencing generated 3,408,285,600 (*R. innominatus*) to 9,832,178,700 (*R. trianthus*) clean reads, with mean coverage from 831 (X) in *R. innominatus* to 1229 (X) in *R. trianthus*. The newly assembled plastome of the eight *Rubus* samples had a quadripartite structure forming a circular molecule ranging from 155,546 bp (*R. trianthus*) to 156,321 bp (*R. lambertianus*) in length (Table 1). The eight cp genomes comprised a large single copy (LSC) region (85,028–85,883 bp) and a small single copy (SSC) region (18,710–18,874 bp), divided by two copies of inverted repeats (IRs) (25,761–25,994 bp) (Fig. 1, Table 1). The guanine-cytosine (GC) content of the eight cp genomes differed slightly, from 37.0% (*R. trianthus* and *R. innominatus*) to 37.3% (*R. coreanus*, *R. hirsutus* and *R. parvifolius*) (Table 1). The GC content of the coding sequence (CDS) ranged from 37.8 to 38.0%. Same as previously reported GC content of *Rubus* in Taiwan, the GC content of IR regions (42.8–42.9%) was higher than LSC (34.8–35.2%) and SSC regions (30.9–31.4%) [31].

**Table 1:** Accession numbers and features of the eight *Rubus* plastomes in the present study

Species	Accession no.	Clean reads	Reads used in assembly	Mean coverage of base (X)	Complete		LSC		SSC		IR		CDS	
					Length (bp)	GC (%)	Length (bp)	GC (%)	Length (bp)	GC (%)	Length (bp)	GC (%)	Length (bp)	GC (%)
*Rubus tephrodes*	MT478113	8,969,158,800	8,367,934	600	156,217	37.2	85,805	35.1	18,830	31.2	25,791	42.8	78,876	38
*R. coreanus*	MT478114	8,545,216,200	8,077,463	620	155,785	37.3	85,028	35.2	18,769	31.3	25,994	42.8	77,404	38
*R. trianthus*	MT478115	9,832,178,700	7,522,556	1229	155,546	37.0	85,305	34.9	18,719	30.9	25,761	42.8	78,838	37.8
*R. lambertianus*	OK127886	3,497,807,100	20,149,705	914	156,321	37.2	85,883	35.1	18,874	31.2	25,782	42.8	78,870	38
*R. hirsutus*	OK127882	3,625,125,600	13,222,835	823	156,020	37.3	85,784	34.8	18,710	31	25,763	42.9	79,212	37.8
*R. parvifolius*	OK127884	3,611,559,900	11,723,986	915	155,906	37.3	85,125	35.2	18,749	31.4	26,016	42.8	78,960	38
*R. hunanensis*	OK127885	3,521,878,500	8,434,742	865	156,217	37.2	85,806	35.1	18,831	31.2	25,790	42.8	78,369	38
*R. innominatus*	OK127883	3,408,285,600	12,384,841	831	155,874	37.0	85,094	35.2	18,795	31.3	25,993	42.8	78,966	38

Note: *GC* guanine-cytosine, *LSC* large single copy, *SSC* small single copy, *IR* inverted repeat, *CDS* coding sequence

Totally, 134 genes were annotated in plastomes of *Rubus tephrodes*, *R. coreanus*, *R. trianthus* and *R. hirsutus*, included 89 CDS, 37 transfer RNA genes (tRNAs), and eight ribosomal RNA (rRNAs). Because *infA* gene were not annotated, four other *Rubus* species (*R. lambertianus*, *R. parvifolius*, *R. innominatus* and *R. hunanensis*) contained 133 genes, including 88 protein-coding genes, eight rRNAs, and 37 tRNAs. Among all, 16 or 18 genes had a double copy in the IR region: seven or five protein-coding genes, seven tRNAs and four rRNAs (Table 1 and Table 2); 17 genes contained one intron (*rps16*, *rpoC1*, *petB*, *petD*, *rpl16*, *rpl22*, *rpl2*, *ndhA*, *ndhB*, and eight tRNA genes) and two genes had two introns (*clpP* and *ycf*3) (Table S1). The *trnK*-UUU gene had the longest intron of 2488–2518 bp in the eight *Rubus* plastomes.

**Table 2:** Gene contents in the cp genomes of *Rubus* species

No.	Group of Genes	Genes Names	Amount
1	Photosystems I	*psaA, psaB, psaC, psaI, psaJ*	5
2	Photosystems II	psbA, psbB, psbC, psbD, psbE, psbF, psbH, psbI, psbJ, psbK, psbL, psbM, psbN, psbT, psbZ, ycf3 **	16
3	Cytochrome b/f complex	petA, petB *, petD *, petG, petL, petN	6
4	ATP synthase	*atpA, atpB, atpE, atpF, atpH, atpI*	6
5	NADH dehydrogenase	ndhA *, ndhB *(×2), ndhC, ndhD, ndhE, ndhF, ndhG, ndhH, ndhI, ndhJ, ndhK	12
6	Rubisco large subunit	*rbcL*	1
7	RNA polymerase	rpoA, rpoB, rpoC1 *, rpoC2	4
8	Ribosomal proteins (SSU)	rps2, rps3, rps4, rps7(×2), rps8, rps11, rps12 (×3), rps14, rps15, rps16 *, rps18, rps19	15
9	Ribosomal proteins (LSU)	rpl2 *(×2), rpl14, rpl16 *, rpl20, rpl22 *, rpl23(× 2), rpl32, rpl33, rpl36	11
10	Assembly/stability of photosystem I	*ycf4*	1
11	Transfer RNAs	*37 tRNAs (6contain an intron, 7 in the IRs)*	37
12	Ribosomal RNAs	*rrn4.5(×2), rrn5(×2), rrn16(× 2), rrn23(× 2)*	8
13	RNA processing	*matK*	1
14	Carbon metabolism	*cemA*	1
15	Cytochrome c synthesis	*ccsA*	1
16	Proteins of unknown function	ycf1 *, ycf2(×2), ycf15(× 2), ycf68	6
17	Other genes	accD, clpP **, infA	3

Note: * Gene contains one intron; ** gene contains two introns; (×2) indicates the number of the repeat unit is 2; (×3) indicates the number of the repeat unit is 3. *infA* were only annotated in *Rubus tephrodes*, *R. coreanus*, *R. trianthus*, *R. hirsutus*

### IR contraction and expansion

**Fig. 2:**
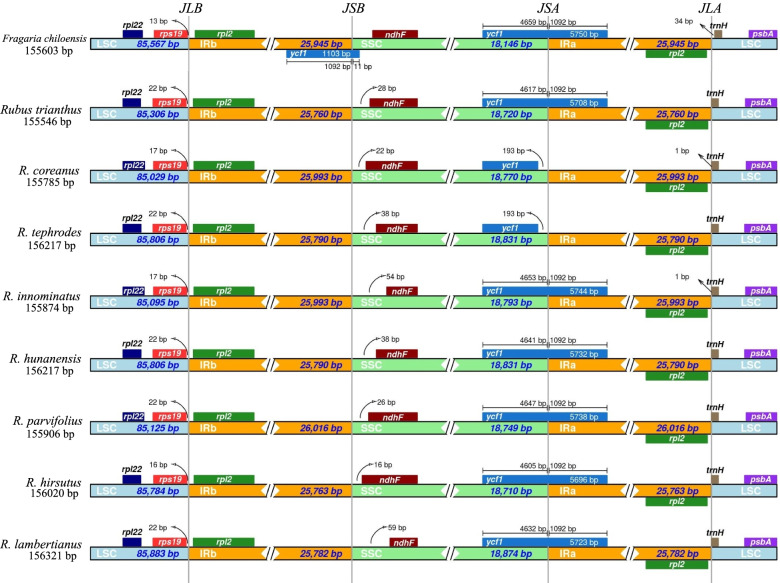
The comparison of four regions (LSC, SSC and two IRs) among twelve cp genome. Numbers above or near the colored genes indicated distances between the gene and the edge of borders. The figure is not in scale for length

Chloroplast genome structures, including the gene content and order, were compared, and analyzed among the eight *Rubus* species (Table 2 and Table S1). The results showed that the eight newly assembled chloroplasts was relatively conserved when concerned in four regions (LSC, SSC and two IRs) boundary (Fig. 2). The lengths of the IR region of the 46 *Rubus* ranged from 25,758–25,993 bp versus 26,238 bp in *Fragaria* (Fig. S1). The LSC-IRb border was located between the genes *rps19* and *rpl2* for ten chroroplast genomes, within the *rps19* gene for *Rubus niveus,* and between the genes *trnH* and *rpl2* for *R. leucanthus*. Three types of SSC-IRa borders were detected among the twelve plastomes. In *R. corchorifolius* and *R. boninensis* the *ycf1* gene was situated in the IRa region, 191 bp and 2 bp apart from the SSC-IRa region, respectively. In *R*. *tephrodes*, *R. niveus* and *R*. *coreanus,* the *ycf1* gene was found entirely in the SSC region and was 0–192 bp away from the SSC-IRa region. For the other seven plastomes, the SSC-IRa border located in the coding region of *ycf1*. The length of the *ycf1* gene range from 4437 bp to 5750 bp and was typically found in the SSC region. The *ndhF* gene located in the SSC region at the SSC-IRb border for all but two species (*R. takesimensis* and *Fragria chiloensis*). The LSC-IRa border was between the *rpl2* and *trnH* genes. The *trnH* gene was found in the LSC region, which has also been reported in dicots [33, 34].

### Comparative Plastome sequence divergence and hotspots regions

**Fig. 3:**
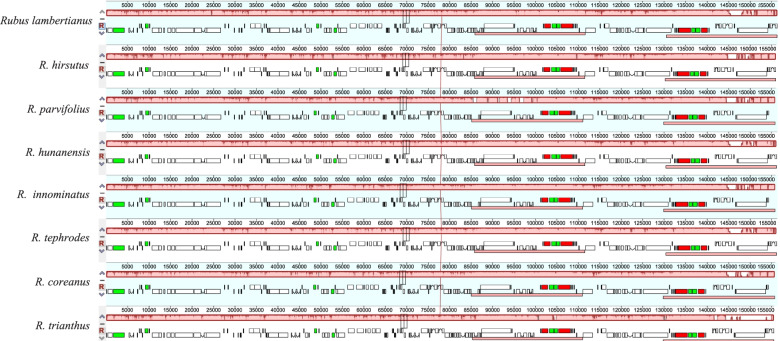
Mauve alignment of eight *Rubus* cp genome revealing no interspecific rearrangements

**Fig. 4:**
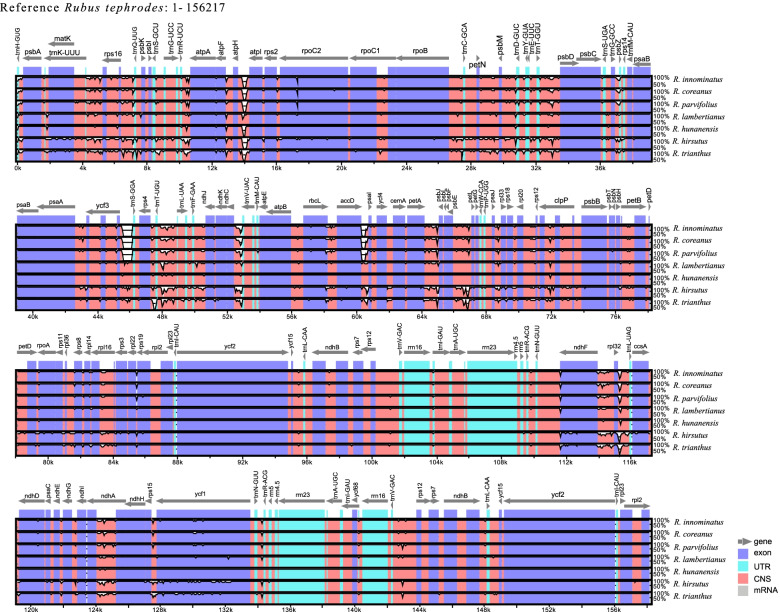
Visualized alignment of the *Rubus* cp genome sequences with annotated *R*. *tephorodes* as reference, using mVISTA. The x-axis represents the base sequence of the alignment and the y-axis represents the pairwise percent identity within 50–100%. Grey arrows represent positions and directions of the genes

Collinearity detection was carried out to analyze and compare the chloroplast genomes. Mauve aligment of plastomes showed that no gene rearrangements within the chloroplast genomes of the eight *Rubus* (Fig. 3). The eight newly assembled chloroplast genomes were compared using the annotated *R. tephrodes* as the reference cp sequence (Fig. 4) to determine interspecific divergence using mVISTA software. The results show that the inverted repeat regions were more stable than the single copy region, and are consistent with those of other studies [25, 35, 36]. The most diverse regions were the intergenic spacers, including *rps16*-*trnQ*, *trnL*-*trnT*, and *rpl32*-*trnL*-*ccsA*.

**Fig. 5:**
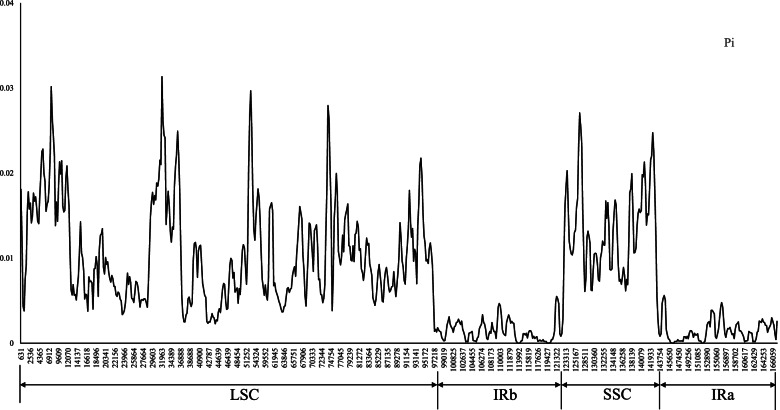
Sliding window analysis of the entire cp genome of eleven *Rubus* species (window length: 600 bp; step size: 200 bp). X-axis: position of the midpoint of a window; Y-axis: nucleotide diversity of each window

The nucleotide variability (Pi) was calculated with the resulting average value of 0.008, and a range of 0 to 0.0313. SSC and LSC were highly variable and IR was relatively conserved (Fig. 5). Nine intergenic regions (*trnK*-*rps16*, *rps16-psbK*, *psbI-trnS*-*trnG*, *trnG*-*atpA*, *petN-psbM-trnD*, *trnE-psbD*, *rps4-trnL*, *petA*-*psbF*, *rpl16*-*rps3*, *ndhF*, *rpl32*-*trnL*-*ccsA* and *ycf1*) were found to be higher variable with Pi values > 0.02, and the first nine fragments were located in the LSC region, while the rest two located in the SSC region. Only two highly variable *trnS*-*trnG* and *ndhF* region (other region used in reference: *rpl16*, *trnL-trnF*, *rbcL*, *rpl20*-*rps12*) has been used to reconstruct the phylogeny of genus *Rubus* to date [18, 19, 21, 22, 37–39]. The highly variable regions detected by comparing entire chloroplast genomes may be useful markers for further phylogenetic study.

### Synonymous (Ks) and nonsynonymous (Ka) substitution rate analysis

**Fig. 6:**
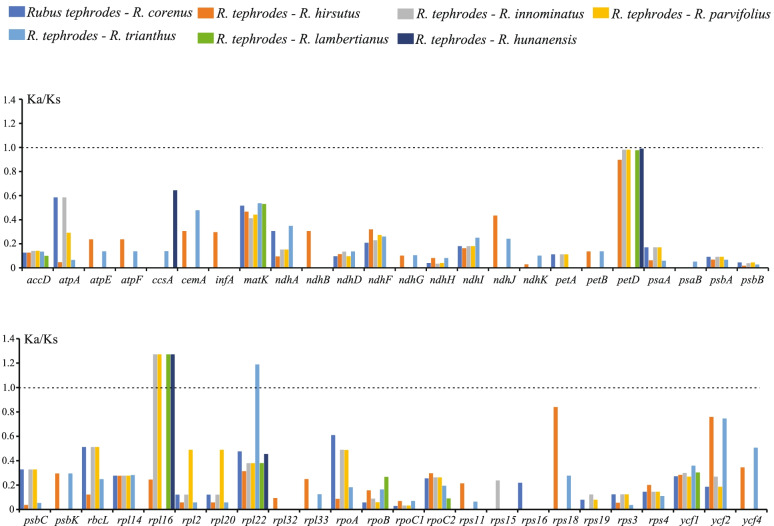
*Ka/Ks* values of protein-coding genes of the seven comparative combinations. *Ka*, nonsynonymous; *Ks*, synonymous

The ratios of nonsynonymous (*Ka*) vs synonymous (*Ks*) substitutions were calculated for shared unique protein coding genes (PCGs) in the eight *Rubus* cp genome, with *R. tephrodes* as the reference (Fig. 6, Table S2). Among 79 shared genes, 31 genes could not be calculated because no variation for identical sequences or without nonsyonymous or synonymous nucleotide substitution. Most of the *Ka/Ks* ratios were less than one, except rpl22 in *R. trianthus* (1.1892), *rpl16* in *R. innominatus* (1.27177), *R. parvifolius* (1.27177), *R. lambertianus* (1.27177) and *R. hunanensis* (1.27177). The results consisted with expected for common sense that the Ka/Ks ratio of most gene is less than one [40]. The above results also indicated the two except genes (*rpl16* and *rpl22*) are undergoing positive selection and some of mutation of the two genes in these species must be advantageous.

### SSR polymorphisms and long repeat structure

**Fig. 7:**
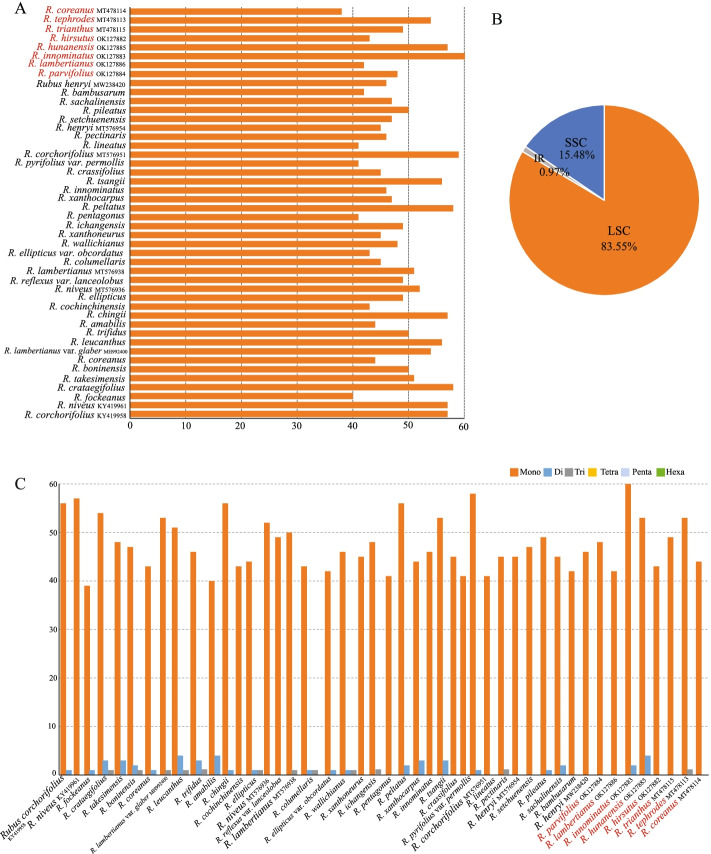
Number and type of simple sequence repeats in the 46 *Rubus* cp genome. **A**, Total number of SSRs detected in each species. **B**, Frequencies of identified SSRs in the large single-copy (LSC), small single-copy (SSC), and inverted repeat (IR) regions. **C**, Numbers and types of SSRs detected in each species

MISA was used to detect the total number of simple sequence repeats (SSRs) in totally 46 *Rubus* plastomes (Fig. 7, Table S3). Totally, 2243 SSRs were found in the 46 plastomes of *Rubus*, of which 3621 SSRs (83.55%) located in the LSC region, 671 SSRs (15.48%) were in the SSC region, and 42 SSRs (.097%) were in the IR region. The number of SSRs detected among the 46 species ranged from 38 (*R. parvifolius*) to 63 (*R. trianthus*) (Table S4). The mononucleotide repeat units were the most identified SSRs. A/T were the most abundant repeats, while AT/TA and AAT/TAA repeats were most found in the dinucleotide and trinucleotide types, respectively. The SSR results showed that A/T repeats were common in the cp genomes, and are consistent with the results of previous studies [35, 41–43]. The SSRs may be potential specific molecular markers to use in genetic diversity and phylogenetic studies for *Rubus* and its related species.

**Fig. 8:**
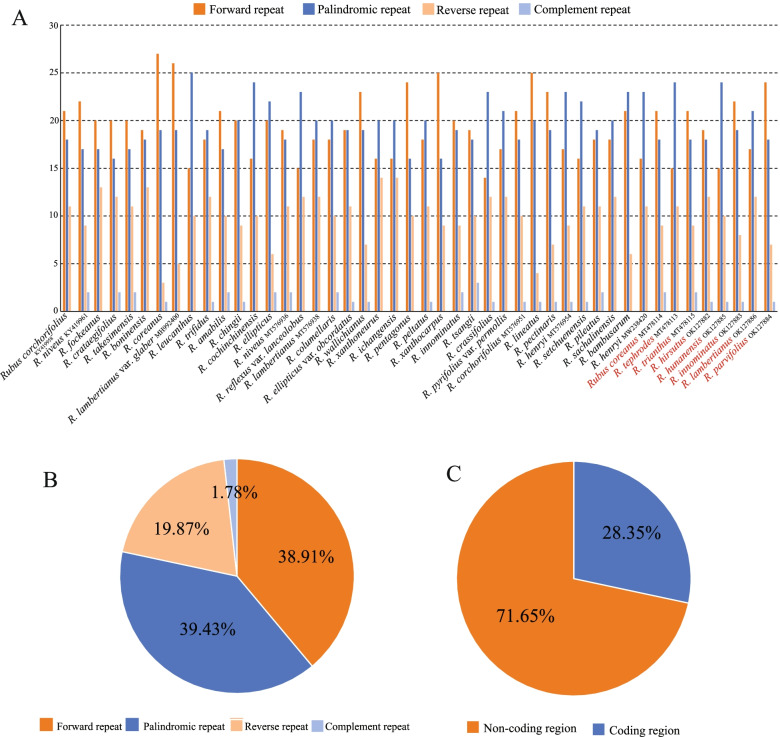
Number and type of long repeat sequences in the 46 *Rubus* cp genome. **A**, Numbers and types of longer repeats in each species. **B**, Frequency of each repeat type. C, Presence of longer repeats in coding regions and non-coding regions

In total, 2300 long repeat structures were identified in the 46 plastomes (Fig. 8, Table S5), which including 895 (38.91%) forward repeats, 907 (39.43%) palindromic repeats, 457 (19.87%) reverse repeats and 41 (1.78%) complement repeats, respectively. Most of these repeats (1648, 71.65%) were distributed in the non-coding regions.

### Phylogenetic analyses

**Fig. 9:**
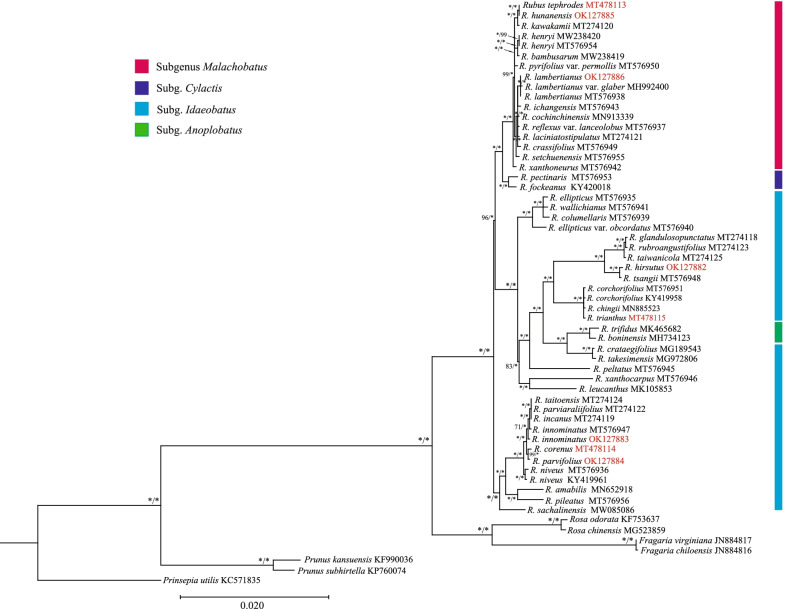
The maximum likelihood (ML) and Bayesian inference (BI) phylogenetic tree based on whole chloroplast genome data. The support values (bootstrap value [BS]/posterior probability [PP]) are indicated at the branches. BS and PP of 100% are indicated by an asterisk

Fifty-one *Rubus* chloroplast genome and other seven plastomes of Rosaceae were used to examine the usability of the chloroplast genome in phylogeny analysis. The maximum likelihood (ML) tree constructed with RaxML and Bayesian inference (BI) tree contructed by MrBayes was topologically congruent and highly supported (Fig. 9). The relationship of the tribe below Rosaceae was congruent with previously reported results [25]. The tree also strongly supported the monophyly of the genus *Rubus*. The *Rubus* clade showed that some species of *Idaeobatus* were likely the original taxa and the polyploidy group may have originated from those primitive species. Other sections of the tree may have evolved from these taxa via different evolutionary events. The main objective of our study was to test the discriminatory power of the chloroplast genome sequences in genus *Rubus*. Additional studies with broader sampling strategies are needed to test the efficiency of the regions identified by our study to clarify the phylogeny of genus *Rubus*.

## Materials and methods

### Material sampling, DNA isolation, and sequencing

Here, eight species, *Rubus tephrodes*, *R. coreanus*, *R. trianthus*, *R. lambertianus*, *R. hirsutus*, *R. parvifolius*, *R. hunanensis* and *R. innominatus* were chosen for sequencing based on their special taxonomic status, their absence or not well assembled in the NCBI. Young, disease-free leaves of wild seedlings were collected and quickly dried with silica gel (Table 3). Voucher specimens were collected for each species and deposited in the herbarium of Huanggang Normal University (formerly, Herbarium of Biology Department of Huanggang Teachers College, HGTC). The specimens were identified by prof. Hongjin Dong (Huanggang Normal University) before deposited. The total genomic DNA was extracted from the leaf tissues using the CTAB method [44] with minor modifications and stored at − 80 °C. The total genomic DNA were sheared into fragments of about 300 bp to construct libraries according to manufacturer’s instructions (Illumina, Hayward, CACA, USA). Sequencing was performed on the Illumina HiSeq 2500 Sequencing System at BGI-Wuhan.

**Table 3:** Sampled species and their voucher specimens used in this study

Species	Voucher Specimen	Coordinate	Location
*Rubus tephrodes* Hance	HGTC HGNU-0024	E 115°47′55.77″, N 31°05′32.68″	China, Hubei, Yingshan
*R. coreanus* Miq.	HGTC HGNU-0194	E 114°47′05.06″, N 29°58′58.17″	China, Hubei, Daye
*R. trianthus* Focke	HGTC HGNU-0301	E 115°51′09.30″, N 30°14′10.36″	China, Hubei, Huangmei
*R. lambertianus* Ser.	HGTC HJD1379	E 115°48′59.05″, N 31°6′54.67″	China, Hubei, Yingshan
*R. hirsutus* Thunb.	HGTC HJD1113	E 114°36′51.64″, N 31°34′16.25”	China, Hubei, Hong’an
*R. parvifolius* L.	HGTC HJD1006	E 115°4′52.01″, N 30°29′46.26”	China, Hubei, Xishui
*R. hunanensis* Hand. - Mazz.	HGTC HJD1069	E 116°1′7.42″, N 30°57′51.44”	China, Hubei, Yingshan
*R. innominatus S.* Moore	HGTC HJD1054	E 116°2′52.85″, N 30°58′20.62”	China, Hubei, Yingshan

### Chloroplast genome assembly and annotation

Raw data with adapter sequences or low-quality sequences was filtered by SOAPnuke software developed by BGI [45]. Then, the high-quality PE reads were used for subsequent analyses. The chloroplast genome was de novo assembled in the GetOrganelle pipeline (http://github.com/Kinggerm/GetOrganelle; [46]). The output graphs file “gfa” was checked in Bandage v. 0.8.1 [47] and the finally sequence paths were selected when the minimum depth of contigs above 100 × and the minimum length > 300 bp. To validate the assembled cp sequence error, raw sequencing reads were mapped to the assembled plastomes using the Bowite2 [48] plug-in in Geneious ver 8.0.2 [48]. The assembled cp genome sequence of the eight *Rubus* samples was annotated using Perl script of PGA [49]. The annotated results were summarized and the final annotations were manually checked using Geneious ver.8.0.2 [48]. The assembly and annotation were completed by mapping the reported plastomes of other well-annotated *Rubus* species. The whole cp sequence with annotated information was submitted to GenBank with accession numbers MT478113-MT478115 and OK127882- OK127886. The physical map of the annotated cp genomes was drawn using the online program OGDRAW [50].

### Comparative Plastome sequence divergence analysis

Gene order comparison of newly-assembled *Rubus* plastomes were performed using the Mauve v.1.0.0 [51] plugin in Geneious v.8.0.2 [48]. We compared the completed plastomes of the eight *Rubus* using mVISTA in Shuffle-LAGAN mode [52] with *R. tephrodes* as the reference. In order to compare the inverted repeated region (IR) contraction or expansion, the detailed information of the boundaries between IR and single copy region (SC) regions were manually obtained in Geneious [48]. The chloroplast genome sequence data sets used for final analysis were aligned using the Windows version of MAFFT [53]. The output data matrix was visualized and manually edited using Geneious [48] or BioEdit [54]. The nucleotide diversity (Pi) of the plastome sequence was calculated using DnaSP v. 6.10 [55], with respect to the whole cp genomes. We used a step size of 200 bp and window length of 800 bp for sliding window analysis.

### Gene selective pressure analysis of eight Rubus cp PCGs

To analysis variation in the evolutionary rates of chloroplast genes, the the Ka_Ks Calculator program Caculator 2.0 was used to calculate the nonsynonymous substitution rates (Ka), synonymous rates (Ks), and their ratios (Ka/Ks). Before calculating, the shared unique protein coding gene sequences (PCGs) was aligned in MEGA [56] (version 10.1.6) by mode of MUSCLE (codons). The gene data matrix was then saved as Clustal (.aln) or Phylip (.phy) format.

### Simple sequence repeats and repeat structure analysis

The Perl script MISA [57] was used to identify microsatellites (mono-, di-, tri-, tetra-, penta-, hexanucleotide repeats), with the following parameters (unit size, min repeats): 10 for mononucleotide, 5 for dinucleotide, 4 for trinucleotide, and 3 for tetra-, penta-, and hexanucleotide. The online REPuter program was used to detect four types of long repeat sequences (forward, reverse, palindromic and complement) in *Rubus* plastomes with a hamming distance of 3 and a minimum repeat size of 30 bp [58].

### Phylogenetic analyses

The newly assembled cp genome of *Rubus* and relative taxa were downloaded from NCBI and then aligned with MAFFT for phylogenetic analysis [53]. Finally, fifty-eight plastomes were used to construct the phylogeny tree (Table S3). RAxML (Version 8 for Windows) was used to run maximum likelihood (ML) analysis [59] with a bootstrap value of 1000. The general time-reversible (GTR) model with a gamma model was used at normal settings to determine the rate of heterogeneity. The Bayesian inference (BI) tree was generated in MrBayes version 3.2 [60] as implemented on the Cyberinfrastructure for Phylogenetic Research (CIPRES) Science Gateway (http://www.phylo.org/, [61]) using the default settings. The best model was determined for each sequence partition, after comparisons among 24 models of nucleotide substitution using jModeltest v.2.1.10 [62]. Figtree v1.4 [63] was used to visualize and adjust the ML trees. The graph generated from Figtree was further revised with Adobe Illustrator (Adobe Systems, Mountain View, CA, USA).

## Conclusions

The complete chloroplast sequences of *Rubus tephrodes*, *R. coreanus,* and *R. trianthus*, *R. lambertianus*, *R. hirsutus*, *R. parvifolius*, *R. hunanensis* and *R. innominatus* of the section *Lampobatus*, and *Idaeobatus* were reported in this study. The comparison analysis of fouty-six *Rubus* plastomes indicated that the structure was relatively conserved. However, the SSC-IR and LSC-IR edges were variable among the chloroplast genomes and the IR region was less varied than the SC region. We identified the location of the SSR sites and highly changeable regions, which may be used as markers in future studies of the *Rubus* species. The ML and BI phylogenetic tree constructed from whole chloroplast sequences illustrated the phylogenetic relationship and was consistent with the results of previous studies. Our results indicate that the whole plastome may be used as a reliable marker in phylogenetic studies of this genus.

## Supplementary Information

**Additional file 1: Table S1**. Genes with introns in the cp genomes of *Rubus* as well as the lengths of the exons and introns.

**Additional file 2: Table S2**. Synonymous (Ks) and nonsynonymous (Ka) analysis of the eight species based on shared unique CDS genes, with Rubus tephrodes as the reference.

**Additional file 3: Table S3**. Taxonomic and accession information for samples used in the study.

**Additional file 4: Table S4**. Statistics of simple sequence repeats in each species of *Rubus. (XLSX 116 kb)*

**Additional file 5: Table S5**. Statistics of longer repeats in each species of *Rubus. (XLSX 94 kb)*

**Additional file 6: Figure S1**. The comparison of four regions (LSC, SSC and two IRs) among twelve cp genome.

## Data Availability

The complete chloroplast genome sequences for the newly assembled eight *Rubus* species are available at GenBank: MT478113-MT478115 and OK127882- OK127886. Raw sequencing reads used in this study were deposited in the GenBank database of Sequence Read Archive (Detailed information was listed in Table S2).
