# Supplementary material for: Complete chloroplast genomes of Rubus species (Rosaceae) and comparative analysis within the genus
Source: BMC Genomics. 2022 Jan 6;23:32. doi: 10.1186/s12864-021-08225-6 (PMC8740016; doi:10.1186/s12864-021-08225-6)
Supplement: Supplementary file 1 — Additional file 1: Table S1. Genes with introns in the cp genomes of Rubus as well as the lengths of the exons and introns. [file 12864_2021_8225_MOESM1_ESM.docx]

**Table S1** Genes with introns in the cp genomes of *Rubus* as well as the lengths of the exons and introns.

|  | **Gene** | **Location** | **Start** | **End** | **ExonI** | **IntronI** | **ExonII** | **IntronII** | **ExonIII** |
| --- | --- | --- | --- | --- | --- | --- | --- | --- | --- |
| *Rubus tephrodes* | trnK-UUU | LSC | 1681 | 4240 | 37 | 2488 | 35 |  |  |
|  | rps16 | LSC | 5260 | 6348 | 39 | 822 | 228 |  |  |
|  | trnS-CGA | LSC | 9067 | 9859 | 32 | 701 | 60 |  |  |
|  | rpoC1 | LSC | 20541 | 23363 | 453 | 768 | 1602 |  |  |
|  | ycf3 | LSC | 43322 | 45322 | 124 | 750 | 230 | 744 | 153 |
|  | trnL-UAA | LSC | 48911 | 49481 | 35 | 486 | 50 |  |  |
|  | trnI-AAU | LSC | 52965 | 53636 | 31 | 580 | 61 |  |  |
|  | clpP | LSC | 71339 | 73420 | 71 | 827 | 294 | 664 | 226 |
|  | petB | LSC | 76375 | 77808 | 6 | 771 | 657 |  |  |
|  | petD | LSC | 78021 | 79220 | 8 | 717 | 475 |  |  |
|  | rpl16 | LSC | 82732 | 84086 | 9 | 947 | 399 |  |  |
|  | rpl22 | LSC | 84880 | 85406 | 394 | 86 | 47 |  |  |
|  | rpl2 | IRB | 85847 | 87346 | 399 | 570 | 531 |  |  |
|  | ndhB | IRB | 96343 | 98555 | 775 | 680 | 758 |  |  |
|  | trnE-UUC | IRB | 103779 | 104803 | 32 | 953 | 40 |  |  |
|  | trnA-UGC | IRB | 104868 | 105750 | 37 | 810 | 36 |  |  |
|  | ndhA | SSC | 121476 | 123818 | 553 | 1251 | 539 |  |  |
|  | ycf1 | SSC | 125787 | 130235 | 1129 | 30 | 3290 |  |  |
|  | trnA-UGC | IRA | 136274 | 137156 | 37 | 810 | 36 |  |  |
|  | trnE-UUC | IRA | 137221 | 138245 | 32 | 953 | 40 |  |  |
|  | ndhB | IRA | 143469 | 145681 | 775 | 680 | 758 |  |  |
|  | rpl2 | IRA | 154678 | 156177 | 399 | 570 | 531 |  |  |

| *R. coreanus* | trnK-UUU | LSC | 1698 | 4275 | 37 | 2506 | 35 |  |  |
| --- | --- | --- | --- | --- | --- | --- | --- | --- | --- |
|  | rps16 | LSC | 5310 | 6384 | 40 | 829 | 206 |  |  |
|  | trnS-CGA | LSC | 9140 | 9931 | 32 | 700 | 60 |  |  |
|  | rpoC1 | LSC | 20522 | 23317 | 432 | 762 | 1602 |  |  |
|  | ycf3 | LSC | 43292 | 45286 | 124 | 729 | 230 | 759 | 153 |
|  | trnL-UAA | LSC | 48277 | 48847 | 35 | 486 | 50 |  |  |
|  | trnI-AAU | LSC | 52426 | 53097 | 31 | 580 | 61 |  |  |
|  | clpP | LSC | 70571 | 72659 | 71 | 822 | 294 | 676 | 226 |
|  | petB | LSC | 75613 | 77046 | 6 | 787 | 641 |  |  |
|  | rpl22 | LSC | 84134 | 84660 | 394 | 86 | 47 |  |  |
|  | rpl2 | IRB | 85075 | 86574 | 391 | 675 | 434 |  |  |
|  | ndhB | IRB | 95571 | 97724 | 721 | 675 | 758 |  |  |
|  | trnE-UUC | IRB | 103224 | 104248 | 32 | 953 | 40 |  |  |
|  | trnA-UGC | IRB | 104313 | 105195 | 37 | 810 | 36 |  |  |
|  | ndhA | SSC | 120883 | 123193 | 553 | 1219 | 539 |  |  |
|  | ycf1 | SSC | 125146 | 129600 | 1129 | 30 | 3296 |  |  |
|  | trnA-UGC | IRA | 135620 | 136502 | 37 | 810 | 36 |  |  |
|  | trnE-UUC | IRA | 136567 | 137591 | 32 | 953 | 40 |  |  |
|  | ndhB | IRA | 143091 | 145244 | 721 | 675 | 758 |  |  |
|  | rpl2 | IRA | 154241 | 155740 | 391 | 675 | 434 |  |  |

| *R. trianthus* | trnK-UUU | LSC | 1689 | 4278 | 37 | 2518 | 35 |  |  |
| --- | --- | --- | --- | --- | --- | --- | --- | --- | --- |
|  | rps16 | LSC | 5319 | 6407 | 39 | 822 | 228 |  |  |
|  | trnS-CGA | LSC | 9042 | 9836 | 32 | 703 | 60 |  |  |
|  | rpoC1 | LSC | 20414 | 23256 | 453 | 788 | 1602 |  |  |
|  | ycf3 | LSC | 43088 | 45075 | 124 | 732 | 230 | 749 | 153 |
|  | trnL-UAA | LSC | 48498 | 49089 | 35 | 507 | 50 |  |  |
|  | trnI-AAU | LSC | 52578 | 53249 | 31 | 580 | 61 |  |  |
|  | clpP | LSC | 70825 | 72916 | 71 | 844 | 294 | 657 | 226 |
|  | petB | LSC | 75873 | 77304 | 6 | 784 | 642 |  |  |
|  | petD | LSC | 77497 | 78700 | 8 | 721 | 475 |  |  |
|  | rpl16 | LSC | 82197 | 83583 | 9 | 979 | 399 |  |  |
|  | rpl22 | LSC | 84329 | 84902 | 395 | 85 | 94 |  |  |
|  | rpl2 | IRB | 85347 | 86846 | 399 | 570 | 531 |  |  |
|  | ndhB | IRB | 95831 | 98043 | 775 | 680 | 758 |  |  |
|  | trnE-UUC | IRB | 103274 | 104298 | 32 | 953 | 40 |  |  |
|  | trnA-UGC | IRB | 104363 | 105245 | 37 | 810 | 36 |  |  |
|  | ndhA | SSC | 120886 | 123220 | 553 | 1243 | 539 |  |  |
|  | trnA-UGC | IRA | 135608 | 136490 | 37 | 810 | 36 |  |  |
|  | trnE-UUC | IRA | 136555 | 137579 | 32 | 953 | 40 |  |  |
|  | ndhB | IRA | 142810 | 145022 | 775 | 680 | 758 |  |  |
|  | rpl2 | IRA | 154007 | 155506 | 399 | 570 | 531 |  |  |

| *R. lambertianus* | trnK-UUU | LSC | 1681 | 4240 | 37 | 2488 | 35 |  |  |
| --- | --- | --- | --- | --- | --- | --- | --- | --- | --- |
|  | rps16 | LSC | 5273 | 6363 | 40 | 821 | 230 |  |  |
|  | trnG-UCC | LSC | 9105 | 9895 | 48 | 720 | 23 |  |  |
|  | rpoC1 | LSC | 20593 | 23416 | 453 | 768 | 1602 |  |  |
|  | ycf3 | LSC | 43403 | 45379 | 126 | 730 | 228 | 748 | 153 |
|  | trnL-UAA | LSC | 48975 | 49545 | 50 | 486 | 35 |  |  |
|  | trnV-UAC | LSC | 53041 | 53712 | 39 | 598 | 35 |  |  |
|  | clpP | LSC | 71408 | 73492 | 71 | 827 | 292 | 667 | 228 |
|  | petB | LSC | 76452 | 77886 | 6 | 787 | 642 |  |  |
|  | petD | LSC | 78099 | 79298 | 8 | 717 | 475 |  |  |
|  | rpl16 | LSC | 82809 | 84163 | 9 | 947 | 399 |  |  |
|  | rpl2 | IRB | 85924 | 87423 | 391 | 675 | 434 |  |  |
|  | ndhB | IRB | 96430 | 98588 | 723 | 680 | 756 |  |  |
|  | rps12 | IRB | 99484 | 100282 | 232 | 541 | 26 |  |  |
|  | trnI-GAU | IRB | 103866 | 104890 | 37 | 953 | 35 |  |  |
|  | trnA-UGC | IRB | 104955 | 105837 | 38 | 810 | 35 |  |  |
|  | ndhA | SSC | 121593 | 123934 | 552 | 1250 | 540 |  |  |
|  | trnA-UGC | IRA | 136368 | 137250 | 38 | 810 | 35 |  |  |
|  | trnI-GAU | IRA | 137315 | 138339 | 37 | 953 | 35 |  |  |
|  | rps12 | IRA | 141923 | 142721 | 232 | 541 | 26 |  |  |
|  | ndhB | IRA | 143617 | 145775 | 723 | 680 | 756 |  |  |
|  | rpl2 | IRA | 154782 | 156281 | 391 | 675 | 434 |  |  |

| *R. hirsutus* | trnK-UUU | LSC | 1680 | 4240 | 37 | 2489 | 35 |  |  |
| --- | --- | --- | --- | --- | --- | --- | --- | --- | --- |
|  | rps16 | LSC | 5257 | 6337 | 40 | 826 | 215 |  |  |
|  | trnG-UCC | LSC | 9141 | 9946 | 23 | 735 | 48 |  |  |
|  | rpoC1 | LSC | 20502 | 23328 | 453 | 770 | 1602 |  |  |
|  | ycf3 | LSC | 43318 | 45292 | 126 | 725 | 228 | 743 | 153 |
|  | trnL-UAA | LSC | 48914 | 49493 | 35 | 495 | 50 |  |  |
|  | trnV-UAC | LSC | 53007 | 53678 | 39 | 598 | 35 |  |  |
|  | clpP | LSC | 71267 | 73350 | 71 | 827 | 292 | 666 | 228 |
|  | petB | LSC | 76322 | 77769 | 6 | 800 | 642 |  |  |
|  | petD | LSC | 77962 | 79171 | 8 | 726 | 475 |  |  |
|  | rpl16 | LSC | 82679 | 84058 | 9 | 972 | 399 |  |  |
|  | rpl2 | IRB | 85831 | 87330 | 391 | 675 | 434 |  |  |
|  | ndhB | IRB | 96323 | 98481 | 723 | 680 | 756 |  |  |
|  | rps12 | IRB | 99377 | 100175 | 232 | 541 | 26 |  |  |
|  | trnI-GAU | IRB | 103762 | 104786 | 37 | 955 | 35 |  |  |
|  | trnA-UGC | IRB | 104851 | 105733 | 38 | 883 | 35 |  |  |
|  | ndhA | SSC | 121336 | 123687 | 552 | 1260 | 540 |  |  |
|  | trnA-UGC | IRA | 136072 | 136954 | 38 | 810 | 35 |  |  |
|  | trnI-GAU | IRA | 137019 | 138043 | 37 | 953 | 35 |  |  |
|  | rps12 | IRA | 141630 | 142428 | 232 | 541 | 26 |  |  |
|  | ndhB | IRA | 143324 | 145482 | 723 | 680 | 756 |  |  |
|  | rpl2 | IRA | 154475 | 155974 | 391 | 675 | 434 |  |  |

| *R. parvifolius* | trnK-UUU | LSC | 1697 | 4269 | 37 | 2501 | 35 |  |  |
| --- | --- | --- | --- | --- | --- | --- | --- | --- | --- |
|  | rps16 | LSC | 5299 | 6443 | 40 | 890 | 215 |  |  |
|  | trnG-UCC | LSC | 9203 | 9992 | 23 | 719 | 48 |  |  |
|  | rpoC1 | LSC | 20639 | 23455 | 453 | 762 | 1602 |  |  |
|  | ycf3 | LSC | 43399 | 45388 | 126 | 728 | 228 | 755 | 153 |
|  | trnL-UAA | LSC | 48379 | 48949 | 35 | 486 | 50 |  |  |
|  | trnV-UAC | LSC | 52493 | 53164 | 39 | 598 | 35 |  |  |
|  | clpP | LSC | 70681 | 72755 | 71 | 824 | 292 | 660 | 228 |
|  | petB | LSC | 75709 | 77143 | 6 | 787 | 642 |  |  |
|  | petD | LSC | 77349 | 78540 | 8 | 709 | 475 |  |  |
|  | rpl16 | LSC | 82063 | 83416 | 9 | 946 | 399 |  |  |
|  | ndhB | IRB | 95666 | 97819 | 723 | 675 | 756 |  |  |
|  | rps12 | IRB | 98715 | 99513 | 232 | 541 | 26 |  |  |
|  | trnI-GAU | IRB | 103343 | 104367 | 37 | 953 | 35 |  |  |
|  | trnA-UGC | IRB | 104432 | 105314 | 38 | 810 | 35 |  |  |
|  | ndhA | SSC | 120990 | 123291 | 552 | 1210 | 540 |  |  |
|  | trnA-UGC | IRA | 135718 | 136600 | 38 | 810 | 35 |  |  |
|  | trnI-GAU | IRA | 136665 | 137689 | 37 | 853 | 35 |  |  |
|  | rps12 | IRA | 141519 | 142317 | 232 | 541 | 26 |  |  |
|  | ndhB | IRA | 143213 | 145366 | 723 | 675 | 756 |  |  |

| *R. hunanensis* | trnK-UUU | LSC | 1681 | 4240 | 37 | 2488 | 35 |  |  |
| --- | --- | --- | --- | --- | --- | --- | --- | --- | --- |
|  | rps16 | LSC | 5260 | 6348 | 40 | 819 | 230 |  |  |
|  | trnG-UCC | LSC | 9068 | 9858 | 23 | 720 | 48 |  |  |
|  | rpoC1 | LSC | 20541 | 23363 | 453 | 768 | 1602 |  |  |
|  | ycf3 | LSC | 43322 | 45322 | 126 | 750 | 228 | 744 | 153 |
|  | trnL-UAA | LSC | 48911 | 49481 | 35 | 486 | 50 |  |  |
|  | trnV-UAC | LSC | 52965 | 53636 | 23 | 720 | 48 |  |  |
|  | clpP | LSC | 71339 | 73420 | 71 | 828 | 292 | 663 | 228 |
|  | petB | LSC | 76375 | 77808 | 6 | 786 | 642 |  |  |
|  | petD | LSC | 78021 | 79220 | 8 | 717 | 475 |  |  |
|  | rpl16 | LSC | 82732 | 84086 | 9 | 947 | 399 |  |  |
|  | rpl2 | IRB | 85847 | 87346 | 391 | 675 | 434 |  |  |
|  | ndhB | IRB | 96343 | 98501 | 723 | 680 | 756 |  |  |
|  | rps12 | IRB | 99397 | 100195 | 232 | 541 | 26 |  |  |
|  | trnI-GAU | IRB | 103779 | 104803 | 37 | 953 | 35 |  |  |
|  | trnA-UGC | IRB | 104868 | 105750 | 38 | 810 | 35 |  |  |
|  | ndhA | SSC | 121476 | 123818 | 552 | 1251 | 540 |  |  |
|  | trnA-UGC | IRA | 136274 | 137156 | 38 | 810 | 35 |  |  |
|  | trnI-GAU | IRA | 137221 | 138245 | 37 | 753 | 35 |  |  |
|  | rps12 | IRA | 141829 | 142627 | 232 | 541 | 26 |  |  |
|  | ndhB | IRA | 143523 | 145681 | 723 | 680 | 756 |  |  |
|  | rpl2 | IRA | 154678 | 156177 | 391 | 675 | 434 |  |  |

| *R. innominatus* | trnK-UUU | LSC | 1690 | 4268 | 37 | 2507 | 35 |  |  |
| --- | --- | --- | --- | --- | --- | --- | --- | --- | --- |
|  | rps16 | LSC | 5279 | 6379 | 40 | 846 | 215 |  |  |
|  | trnG-UCC | LSC | 9137 | 9926 | 23 | 719 | 48 |  |  |
|  | rpoC1 | LSC | 20566 | 23382 | 453 | 762 | 1602 |  |  |
|  | ycf3 | LSC | 43328 | 45318 | 126 | 730 | 228 | 754 | 153 |
|  | trnL-UAA | LSC | 48359 | 48929 | 35 | 486 | 50 |  |  |
|  | trnV-UAC | LSC | 52481 | 53152 | 39 | 598 | 35 |  |  |
|  | clpP | LSC | 70654 | 72727 | 71 | 822 | 292 | 661 | 228 |
|  | petB | LSC | 75681 | 77116 | 6 | 788 | 642 |  |  |
|  | petD | LSC | 77322 | 78513 | 8 | 709 | 475 |  |  |
|  | rpl16 | LSC | 82037 | 83388 | 9 | 944 | 399 |  |  |
|  | ndhB | IRB | 95636 | 97789 | 723 | 675 | 756 |  |  |
|  | rps12 | IRB | 98685 | 99483 | 232 | 541 | 26 |  |  |
|  | trnI-GAU | IRB | 103289 | 104313 | 37 | 953 | 35 |  |  |
|  | trnA-UGC | IRB | 104378 | 105260 | 38 | 810 | 35 |  |  |
|  | ndhA | SSC | 120964 | 123275 | 552 | 1220 | 540 |  |  |
|  | trnA-UGC | IRA | 135708 | 136590 | 38 | 810 | 35 |  |  |
|  | trnI-GAU | IRA | 136655 | 137679 | 37 | 952 | 35 |  |  |
|  | rps12 | IRA | 141485 | 142283 | 232 | 541 | 26 |  |  |
|  | ndhB | IRA | 143179 | 145332 | 723 | 675 | 756 |  |  |
