# Supplementary figures and images for: Complete chloroplast genomes of Rubus species (Rosaceae) and comparative analysis within the genus
Source: BMC Genomics. 2022 Jan 6;23:32. doi: 10.1186/s12864-021-08225-6 (PMC8740016; doi:10.1186/s12864-021-08225-6)

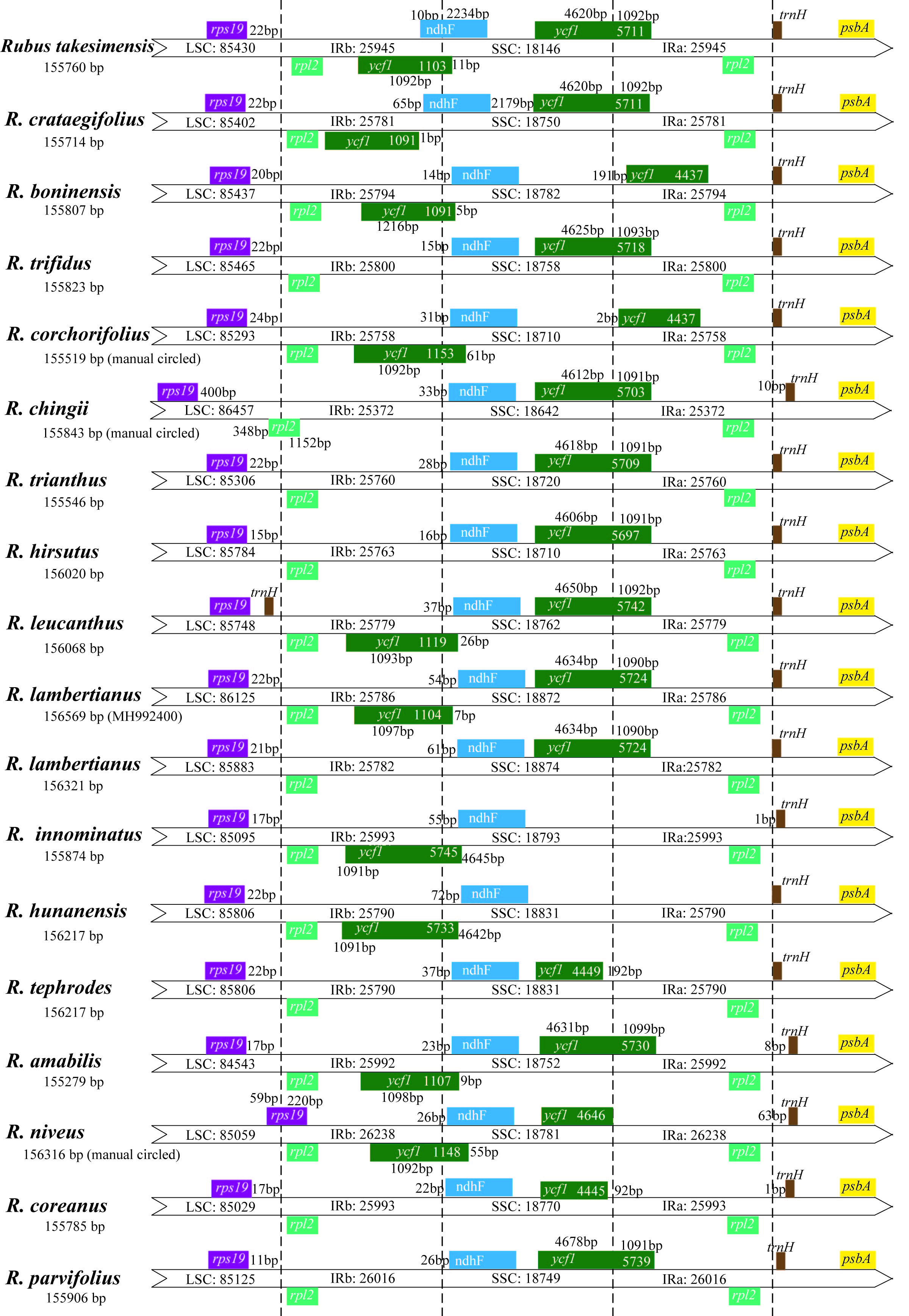

Supplement: Supplementary file 6 — Additional file 6: Figure S1. The comparison of four regions (LSC, SSC and two IRs) among twelve cp genome. [file 12864_2021_8225_MOESM6_ESM.jpg]
